# Impairments in cognition and neural precursor cell proliferation in mice expressing constitutively active glycogen synthase kinase-3

**DOI:** 10.3389/fnbeh.2015.00055

**Published:** 2015-03-04

**Authors:** Marta Pardo, Margaret K. King, Emma Perez-Costas, Miguel Melendez-Ferro, Ana Martinez, Eleonore Beurel, Richard S. Jope

**Affiliations:** ^1^Departments of Psychiatry and Behavioral Sciences and Biochemistry and Molecular Biology, Miller School of Medicine, University of MiamiMiami, FL, USA; ^2^Department of Psychiatry, University of Alabama at BirminghamBirmingham, AL, USA; ^3^Centro de Investigaciones Biologicas-CSICMadrid, Spain

**Keywords:** glycogen synthase kinase-3, neurogenesis, novel object recognition, spatial memory, environmental enrichment

## Abstract

Brain glycogen synthase kinase-3 (GSK3) is hyperactive in several neurological conditions that involve impairments in both cognition and neurogenesis. This raises the hypotheses that hyperactive GSK3 may directly contribute to impaired cognition, and that this may be related to deficiencies in neural precursor cells (NPC). To study the effects of hyperactive GSK3 in the absence of disease influences, we compared adult hippocampal NPC proliferation and performance in three cognitive tasks in male and female wild-type (WT) mice and GSK3 knockin mice, which express constitutively active GSK3. NPC proliferation was ~40% deficient in both male and female GSK3 knockin mice compared with WT mice. Environmental enrichment (EE) increased NPC proliferation in male, but not female, GSK3 knockin mice and WT mice. Male and female GSK3 knockin mice exhibited impairments in novel object recognition, temporal order memory, and coordinate spatial processing compared with gender-matched WT mice. EE restored impaired novel object recognition and temporal ordering in both sexes of GSK3 knockin mice, indicating that this repair was not dependent on NPC proliferation, which was not increased by EE in female GSK3 knockin mice. Acute 1 h pretreatment with the GSK3 inhibitor TDZD-8 also improved novel object recognition and temporal ordering in male and female GSK3 knockin mice. These findings demonstrate that hyperactive GSK3 is sufficient to impair adult hippocampal NPC proliferation and to impair performance in three cognitive tasks in both male and female mice, but these changes in NPC proliferation do not directly regulate novel object recognition and temporal ordering tasks.

## Introduction

Cognitive deficits constitute a critical pathological feature that is difficult to treat in many psychiatric and neurological disorders, which have been suggested to be associated with impaired adult hippocampal neurogenesis (Leuner et al., 2006; Massa et al., 2011; King et al., 2014). Neurogenesis involves neural precursor cell (NPC) proliferation and differentiation into neurons, a process that has been linked to hippocampal-dependent cognitive processes (Kempermann, 2002; van Praag et al., 2005; Leuner et al., 2006; Deng et al., 2010; Massa et al., 2011). The potential coexistence of impairments in cognitive processes and hippocampal neurogenesis, for example in Alzheimer’s disease (Mu and Gage, 2011), Fragile X syndrome (Guo et al., 2012), and mood disorders (Jacobs et al., 2000), raises the possibility that the two are linked. Thus, impaired neurogenesis in multiple disorders may contribute to cognitive deficits, and bolstering neurogenesis may provide a mechanism to improve learning and memory (Bruel-Jungerman et al., 2005; Jessberger et al., 2009).

The serine/threonine kinase glycogen synthase kinase-3 (GSK3) is a critical regulator of both cognition (King et al., 2014) and neurogenesis (Kim et al., 2009). GSK3 refers to two isoforms, GSK3α and GSK3β, that are primarily regulated by inhibitory phosphorylation on Ser21-GSK3α and Ser9-GSK3β (Jope and Johnson, 2004). The importance of inhibitory control of GSK3 can be studied using GSK3α21A/21A/β9A/9A knockin mice (hereafter referred to as GSK3 KI mice), with the regulatory serines of both GSK3 isoforms mutated to alanines (McManus et al., 2005; Eom and Jope, 2009; Polter et al., 2010). These mutations maintain GSK3 maximally active within the physiological range, since both GSK3 isoforms are expressed at normal levels. Conversely, inhibition of GSK3 in healthy wild-type (WT) rodents generally has little effect on performance on cognitive tasks, but ameliorates cognitive impairments associated with a wide variety of injury and disease models in rodents (King et al., 2014). However, these effects of GSK3 inhibitors often are apparently due to actions involving attenuation of responses to the insult (King et al., 2014), leaving open the question of whether hyperactive GSK3 alone is sufficient to impair cognition. Abundant evidence has shown that GSK3 modulates adult hippocampal NPC proliferation, which is severely impaired in male GSK3 KI mice (Eom and Jope, 2009) and is increased by molecular deletion or inhibition of GSK3 (Chen et al., 2000; Hashimoto et al., 2003; Silva et al., 2008; Wexler et al., 2008; Kim et al., 2009; Morales-Garcia et al., 2012). These findings raise the hypothesis that hyperactive GSK3 may cause cognitive impairments and that these may be associated with impaired NPC proliferation.

Like GSK3, environmental enrichment (EE) also affects both cognition and components of neurogenesis in rodents. NPC proliferation is increased in male rodents exposed to EE or exercise, a key component of EE (Kempermann et al., 1997, 2002; Brown et al., 2003; Komitova et al., 2005; Rossi et al., 2006; Leal-Galicia et al., 2007; Zhao et al., 2008; Hu et al., 2010; Chakrabarti et al., 2011; Mustroph et al., 2012; Salmaso et al., 2012; Tanti et al., 2013). Unlike male mice, EE did not alter NPC proliferation in the hippocampus of female mice (Kempermann et al., 1997; van Praag et al., 1999; Brown et al., 2003; Westenbroek et al., 2004; Kobilo et al., 2011), supporting substantial evidence that male and female rodents differ in mechanisms influencing both cognition and NPC proliferation (Barha and Galea, 2010; Chow et al., 2013; Galea et al., 2013). EE has also been shown in many studies to increase performance of rodents in a multitude of cognitive tasks (Sale et al., 2014), including novel object recognition (O’Callaghan et al., 2007; Leal-Galicia et al., 2008; Doulames et al., 2014) and spatial processing (Schrijver et al., 2004; Doulames et al., 2014). Thus, EE may improve both cognition and NPC proliferation, raising the possibilities that these outcomes are linked and that EE may counteract impairments in these processes caused by hyperactive GSK3. Therefore, we used GSK3 KI mice to examine if hyperactive GSK3 is sufficient to impair hippocampal NPC proliferation and performance on three cognitive tasks: novel object recognition (Clark et al., 2000; Hunsaker et al., 2008; Antunes and Biala, 2012), a temporal order task (Kesner et al., 2004, 2005; Hunsaker et al., 2006), and a coordinate spatial processing task (Goodrich-Hunsaker et al., 2008; Hunsaker et al., 2008, 2012). We also tested if there were differences in male and female mice and if EE influenced these characteristics.

## Materials and methods

### Mice

**Figure 1:**
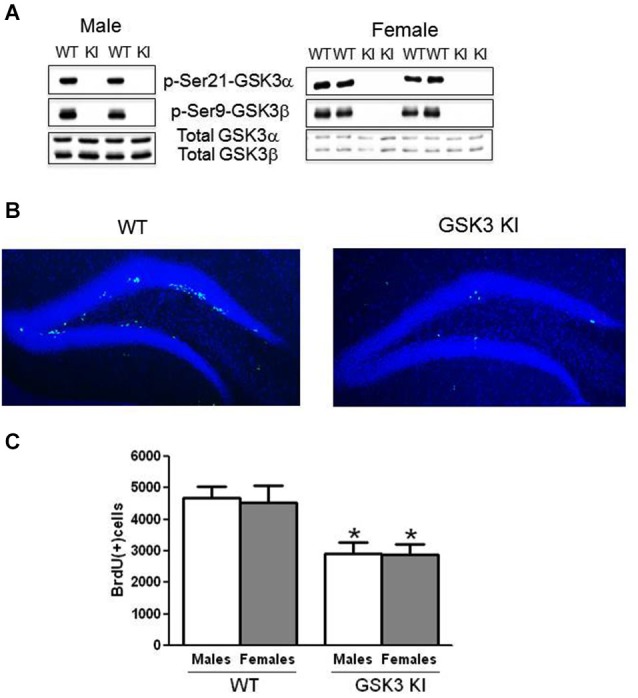
**NPC proliferation is impaired in the dentate gyrus of GSK3 knockin mice. (A)** Immunoblots showing the absence of serine phosphorylation of GSK3α and GSK3β in the hippocampus of male GSK3 knockin (KI) mice (left panel), as we previously reported (Eom and Jope, 2009), and female GSK3 KI mice (right panel). Total levels of GSK3α and GSK3β are equal in GSK3 KI and wild-type (WT) mice. **(B)** Immunohistochemical detection of BrdU-positive cells (green) in the hippocampus of wild-type (WT) and GSK3 knockin (KI) mice. Nuclei are labeled with bisbenzimide (blue). **(C)** Unbiased stereological quantitation of BrdU-positive cells in the hippocampal dentate gyrus of male and female WT and GSK3 KI mice. NPC proliferation significantly differed between WT males (4685 ± 353) and GSK3 KI males (2906 ± 382) (*t*_(20)_ = 3.42, *p* < 0.01) and between WT females (4543 ± 519) and GSK3 KI females (2884 ± 337) (*t*_(14)_ = 2.28, *p* < 0.05). Two-way ANOVA (genotype × sex) followed by Bonferroni’s multiple comparison test; main effect for genotype: *F*_(1,34)_ = 15.585, *p* < 0.001. Values are means ± S.E.M.; *p < 0.05 compared to WT control mice of the same gender.

Male and female adult (9–11 weeks old at the time of behavioral testing and NPC proliferation analysis) homozygous GSK3α/β^21*A*/21*A*/9*A*/9*A*^ KI mice and matched WT mice were used (McManus et al., 2005). Inhibitory serine-phosphorylation of GSK3α and GSK3β is absent in these mice, whereas the total levels of both GSK3 isoforms are equivalent to WT mice (Figure 1A). GSK3 KI mice develop and reproduce normally with no overt phenotype (McManus et al., 2005; Eom and Jope, 2009; Polter et al., 2010). Mice were housed in groups of 3–5 in standard cages in light and temperature controlled rooms and were treated in accordance with NIH and the University of Miami Institutional Animal Care and Use Committee regulations. Mice were treated intraperitoneally (i.p.) with vehicle or thiadiazolidindione-8 (TDZD-8; 5 mg/kg), a selective non-ATP competitive inhibitor of GSK3 (Martinez et al., 2002), dissolved in 5% Tween80 and 5% DMSO in saline, 1 h prior to behavioral testing. For EE, mice were housed in a large cage (55 cm × 32 cm × 22 cm) with extra wood chip bedding, nesting material, and a variety of sized, shaped, and colored objects for 25 days. Weekly the objects were washed and moved, and new objects were added.

### Immunoblot analysis

Mouse hippocampi were rapidly dissected in ice-cold phosphate-buffered saline. Brain regions were homogenized in ice-cold lysis buffer containing 20 mM Tris-HCl, pH 7.4, 150 mM NaCl, 2 mM EDTA, 1% Triton X-100, 10% glycerol, 1 µg/ml leupeptin, 1 µg/ml aprotinin, 1 µg/ml pepstatin, 1 mM phenylmethanesulfonyl fluoride, 50 mM NaF, 1 mM sodium orthovanadate, and 100 nM okadaic acid. The lysates were centrifuged at 20,800 × g for 10 min. Protein concentrations in the supernatants were determined using the Bradford protein assay (Bradford, 1976). Lysates were mixed with Laemmli sample buffer (2% SDS) and placed in a boiling water bath for 5 min. Proteins (10 µg) were resolved in SDS-polyacrylamide gels, transferred to nitrocellulose, and incubated with primary antibodies to phospho-Ser9-GSK3β (1:1000; Cell Signaling Technology), phospho-Ser21-GSK3α (1:1000; Cell Signaling Technology), and total GSK3α/β (1:2000; Millipore). Immunoblots were developed using horseradish peroxidase-conjugated goat anti-mouse, or goat anti-rabbit IgG, followed by detection with enhanced chemiluminescence.

### Measurement of hippocampal NPC proliferation

5-Bromo-2′-deoxyuridine (BrdU; 100 mg/kg; Sigma-Aldrich, St Louis, MO) was administered i.p. three times at 2 h intervals, and mice were analyzed 24 h later, as we previously described (Eom and Jope, 2009). Mice were anesthetized and transcardially perfused with 0.9% NaCl followed by 4% paraformaldehyde in 0.1 M phosphate buffer (pH 7.4). Brains were post-fixed overnight in 4% paraformaldehyde at 4°C and cryoprotected in 30% sucrose/phosphate buffered saline (PBS). Each brain was sliced coronally (30 µm) with a microtome (Leica, Nubloch, Germany) through the rostrocaudal hippocampus and stored in PBS with 0.01% sodium azide. Every sixth section was analyzed for BrdU-specific immunohistochemistry as previously described (Eom and Jope, 2009). Sections were washed in 0.05 M Tris-HCl buffer (TBS, pH 7.4) and incubated in 1 N HCl on ice for 10 min, in 2 N HCl for 10 min at room temperature, and in 2 N HCl at 37°C for 20 min, washed with 1 M borate buffer, pH 8.5, on ice, and rinsed in TBS. The sections were incubated with anti-BrdU antibody (1:500; BU1/75; Abcam) in 15% normal goat serum and TBS blocking buffer (1% bovine serum albumin, 0.2% TritonX100 in TBS) for 20 h at 4°C. Sections were washed with TBS and incubated with Alexa Fluor 488 goat anti-rat (1:200, Invitrogen) in 10% normal goat serum and TBS blocking buffer for 2 h at room temperature in the dark. Cell nuclei were stained by incubating sections for 5 min in 0.2 µg/ml bisbenzimide (Hoechst 33258; Sigma). BrdU positive cells in the granule cell layer of the dentate gyrus and the subgranular zone were counted in each section and analyzed by unbiased stereology using the StereoInvestigator system (MicroBrightField, Williston, VT). To distinguish single cells within clusters, all counts were performed using a 63× oil immersion objective (Olympus BX-51), omitting cells in the outermost focal plane. The total number of BrdU-labeled cells per section was determined and multiplied by 6 to obtain the total number of cells per dentate gyrus. The parameters for the steorological study were the following: Section fraction 6; grid size 250 × 90 µm; counting frame 40 × 40 µm; CE values were close to 0.1 in all experiments; previous analyses in biological structures have indicated that a CE near 0.1 is adequate to find a real difference in the number of objects counted between two different samples (Gundersen and Jensen, 1987; West, 1993).

### Behavioral analyses

For all behavioral assessments, mice were acclimated to the room containing the behavioral instruments for 30 min before testing, the sessions were filmed, a white noise generator (55 dB) was used, and each apparatus and object was cleaned with 70% ethanol between each test session. Measures of novel object recognition, temporal ordering for visual objects (referred to as temporal order task), and coordinate spatial processing, were assessed by published methods (King and Jope, 2013; Franklin et al., 2014). Behavioral tests were conducted during 3 consecutive days, one test every 24 h. For all cognitive assessments, time spent exploring an object included the mouse sniffing or touching the object with its nose, vibrissa, mouth, or forepaws. Time spent near or standing on top of an object without interacting with it was not counted as exploration.

The novel object recognition task was carried out in a four-step procedure with male mice as previously described (Hoge and Kesner, 2007; Hunsaker et al., 2012; Franklin et al., 2014) and a two-step procedure for female mice because female WT mice had difficulty with the four-step procedure (see Section Discussion). For male mice, a Plexiglass box (26 cm long × 20 cm wide × 16 cm tall) and four objects in duplicate (4–6 cm diameter × 2–6 cm height) were used. Each mouse was allowed to explore two identical copies of Object 1 for 5 min, rested for 5 min in an opaque holding container, and then allowed to explore two copies of Object 2 for 5 min, followed by the same protocol for Object 3. For the test phase, each mouse was allowed to explore an unused copy of Object 1 and a novel Object 4 for 5 min. More time spent exploring the novel Object 4 than the familiar Object 1 indicates normal memory processing. Time spent exploring each object was obtained from videos, and the exploration ratio was calculated as the times (exploring Object 4 − exploring Object 1)/(exploring Object 1 plus Object 4). For female mice, the procedure was identical to that described above except after exploring Object 1, mice were exposed to an unused copy of Object 1 and a novel Object 2 for 5 min for the test phase.

For the temporal order task, each mouse underwent three sessions to explore three new sets of objects (Objects 5, 6, 7). During the test session, the mouse was allowed to explore an unused copy of Object 5 and an unused copy of Object 7 for 5 min. Normal temporal order memory is exhibited by mice spending more time exploring the first object presented (Object 5) than the most recent object presented (Object 7). The exploration ratio was calculated as time (exploring Object 5 − exploring Object 7)/(exploring Object 5 plus Object 7).

For the coordinate spatial processing task, during the habituation stage each mouse was allowed to explore two novel objects that were 45 cm apart for 15 min. After 5 min in an opaque holding container, each mouse was allowed to explore for 5 min the same two objects that had been moved closer together (30 cm). Mice that remember the distance between objects display increased exploration of the objects during the test session compared with the last 5 min of the habituation phase. The exploration ratio was calculated as time (exploring during the 5 min test session)/(exploring during the 5 min test session plus the last 5 min of the habituation session).

### Statistical analyses

Results were analyzed by one-way ANOVA with condition (genotype × treatment), two-way ANOVA with condition (genotype × sex), or three-way ANOVA with condition (genotype × sex × treatment) followed by Dunnett’s test and Bonferroni’s multiple comparison tests, or by Student’s *t*-test where indicated.

## Results

### Adult hippocampal NPC proliferation is impaired in male and female GSK3 KI mice

Immunohistochemical analysis of BrdU-labeled cells demonstrated a predominant location in the subgranular zone of the dentate gyrus in both WT and GSK3 KI mice (Figure 1B). We previously reported that WT and GSK3 KI mouse brains displayed equivalent morphological features, hippocampal volumes, and staining for neuronal nuclei (NeuN) and glial fibrillary acidic protein (GFAP; Eom and Jope, 2009). As we reported previously (Eom and Jope, 2009), quantitative unbiased stereology analysis revealed that the number of BrdU-labeled cells within the dentate gyrus in male GSK3 KI mice was significantly 40% lower than in matched WT mice (WT males: 4685 ± 353, GSK3 KI males: 2906 ± 382; *t*_(20)_ = 3.42, *p* < 0.01) (Figure 1C). We extended the analysis to female mice, which revealed a 40% deficit in NPC proliferation in female GSK3 KI mice compared with female WT mice (WT females: 4543 ± 519, GSK3 KI females: 2884 ± 338; *t*_(14)_ = 2.28, *p* < 0.05). Two-way ANOVA condition (genotype × sex) revealed a main effect for genotype (*F*_(1,34)_ = 15.585, *p* < 0.001). There was no main effect for sex (*F*_(1,34)_ = 0.036, *p* = 0.851), and there was no interaction between male and female WT mice or between male and female GSK3 KI mice (*F*_(1,34)_ = 0.019, *p* = 0.89).

### Cognition is impaired in male and female GSK3 KI mice

**Figure 2:**
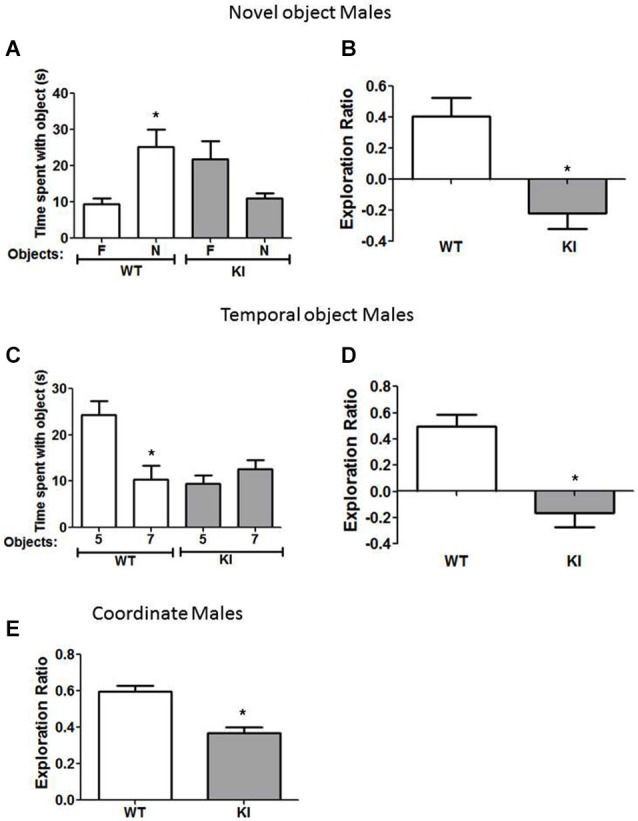
**Cognition is impaired in male GSK3 KI mice. (A,B)** Performance of male wild-type (WT) (*n* = 10) and GSK3 KI (*n* = 12) mice on novel object recognition. **(A)** Time spent exploring the novel (N) and familiar (F) object. (Student’s *t*-test, *p < 0.05 compared to time spent with familiar object). **(B)** Exploration ratio (Student’s *t*-test, *p < 0.05 compared to WT mice). **(C,D)** Performance of male WT (*n* = 10) and GSK3 KI (*n* = 12) mice on the temporal order task. **(C)** Time spent exploring the first (5) and last (7) object presented. (Student’s *t*-test, *p < 0.05 compared to time spent with object 5). **(D)** Exploration ratio (Student’s *t*-test, *p < 0.05 compared to WT mice). **(E)** Performance of male WT (*n* = 10) and GSK3 KI (*n* = 12) mice on coordinate spatial processing task (Student’s *t*-test, *p < 0.05 compared to WT mice).

We tested if male and female GSK3 KI mice displayed altered performance in three cognitive tasks compared with gender-matched WT mice: novel object recognition, temporal order memory, and coordinate spatial processing. In the novel object recognition test (Figure 2A), a measure of recognition memory, male WT mice spent significantly more time exploring the novel than the familiar object (25 ± 5 vs. 9 ± 2 s; *t*_(18)_ = 3.14, *p* < 0.01). In contrast, male GSK3 KI mice did not display preference for the novel object (11 ± 1 vs. 22 ± 5 s; *t*_(22)_ = 2.057, *p* = 0.052). The exploration ratio (Figure 2B) was significantly different between male WT mice (0.41 ± 0.12) and GSK3 KI mice (−0.22 ± 0.10; *t*_(20)_ = 3.84, *p* < 0.01).

In the temporal order task (Figure 2C), a task used to assess episodic-like memory, male WT mice spent significantly more time exploring the first object presented, indicative of memory of the order the objects were presented (Object 5: 24 ± 3 vs. Object 7: 10 ± 3 s; *t*_(18)_ = 3.23, *p* < 0.01). Male GSK3 KI mice displayed impaired ability to discriminate the presentation order, as they did not exhibit significant differences in time spent exploring each object (Object 5: 9 ± 2 vs. Object 7: 13 ± 2 s; *t*_(22)_ = 1.17, *p* = 0.25). The exploration ratio (Figure 2D) was significantly different between male WT mice (0.49 ± 0.09) and GSK3 KI mice (−0.17 ± 0.11; *t*_(20)_ = 4.53, *p* < 0.01).

In the coordinate spatial processing task (Figure 2E), a task developed for the measurement of spatial memory, male WT mice, but not male GSK3 KI mice, spent more time exploring objects during the testing period when the distance between objects was changed compared to the last 5 min of the habituation phase. Thus, there was a significant effect of genotype on the exploration ratio (WT: 0.60 ± 0.03: KI: 0.37 ± 0.03; *t*_(20)_ = 5.34, *p* < 0.01).

**Figure 3:**
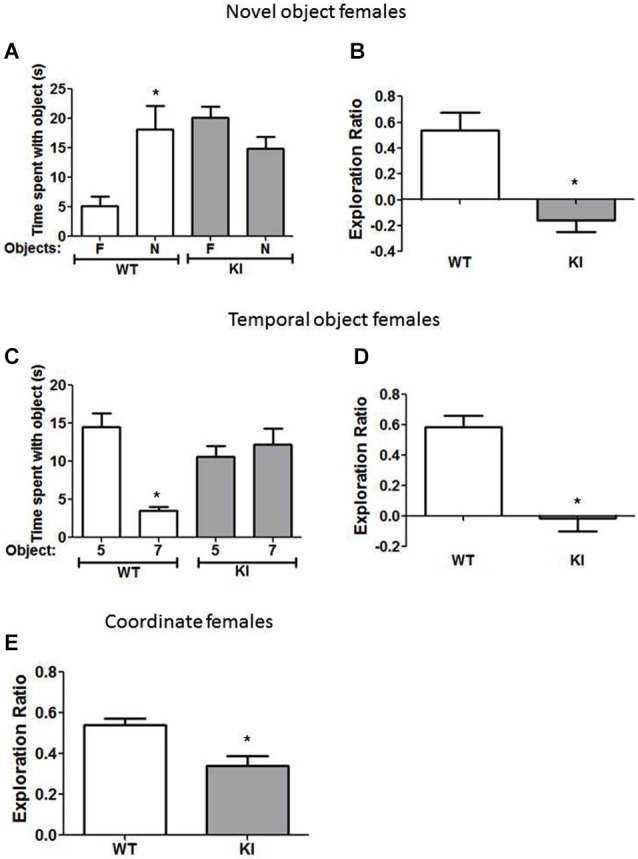
**Cognition is impaired in female GSK3 KI mice. (A,B)** Performance of female wild-type (WT) (*n* = 10) and GSK3 KI (*n* = 9) mice on novel object recognition. **(A)** Time spent exploring the novel (N) and familiar (F) object. (Student’s *t*-test, *p < 0.05 compared to time spent with familiar object). **(B)** Exploration ratio (Student’s *t*-test, *p < 0.05 compared to WT mice). **(C,D)** Performance of female WT (*n* = 10) and GSK3 KI (*n* = 10) mice on the temporal order task. **(C)** Time spent exploring the first (5) and last (7) object presented. (Student’s *t*-test, *p < 0.05 compared to time spent with object 5). **(D)** Exploration ratio (Student’s *t*-test, *p < 0.05 compared to WT mice). **(E)** Performance of female WT (*n* = 24) and GSK3 KI (*n* = 10) mice on coordinate spatial processing task (Student’s *t*-test, *p < 0.05 compared to WT mice).

We also tested if female GSK3 KI mice displayed cognitive deficits similar to male GSK3 KI mice. In the novel object recognition task (Figure 3A), female WT mice spent significantly more time exploring the novel than the familiar object (18 ± 4 vs. 5 ± 2 s; *t*_(18)_ = 3.02, *p* < 0.05). In contrast, female GSK3 KI mice did not display preference for the novel object (15 ± 3 vs. 20 ± 2 s; *t*_(16)_ = 1.89, *p* = 0.08). The exploration ratio (Figure 3B) was significantly different between female WT mice (0.54 ± 0.14) and female GSK3 KI mice (−0.16 ± 0.09; *t*_(17)_ = 4.07, *p* < 0.01).

In the temporal object task (Figure 3C), female WT mice spent significantly more time exploring the first object presented (Object 5: 15 ± 2 vs. Object 7: 4 ± 1 s; *t*_(18)_ = 5.74, *p* < 0.01). However, female GSK3 KI mice spent equivalent times exploring each object (Object 5: 11 ± 1 vs. Object 7: 12 ± 2 s; *t*_(18)_ = 0.64, *p* = 0.53). The exploration ratio (Figure 3D) was significantly different between female WT mice (0.58 ± 0.08) and female GSK3 KI mice (−0.01 ± 0.09; *t*_(18)_ = 5.19, *p* < 0.01).

In the coordinate spatial processing task (Figure 3E), female WT mice, but not female GSK3 KI mice, spent more time exploring objects during the testing period when the distance between objects was changed compared to the last 5 min of the habituation phase. Thus, there was a significant difference between genotypes in the exploration ratio (WT: 0.54 ± 0.03: KI: 0.34 ± 0.05; *t*_(32)_ = 3.28, *p* < 0.01).

Overall, we found that constitutively active GSK3 in both male and female GSK3 KI mice caused significantly impaired performance in the novel object recognition task, the temporal order task, and coordinate spatial processing.

### Modulation of GSK3 and hippocampal NPC proliferation by EE

**Figure 4:**
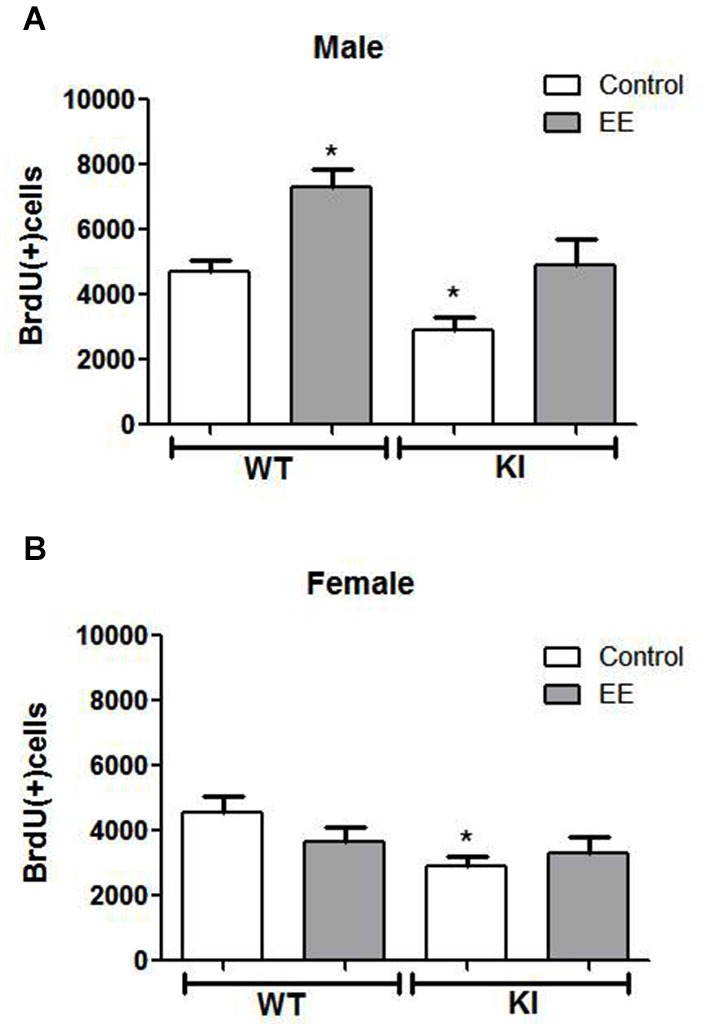
**Effects of environmental enrichment (EE) on NPC proliferation in the dentate gyrus**. Unbiased stereological quantitation of BrdU-positive cells in the hippocampal dentate gyrus of male and female wild-type (WT) (control males *n* = 11, EE males *n* = 9, control females *n* = 10, EE females *n* = 10) and GSK3 knockin (KI) (control males *n* = 11, EE males *n* = 5, control females *n* = 6, EE females *n* = 9) mice with and without 25 days of EE. Three-way ANOVA (condition: genotype × sex × treatment) revealed a main effect for sex: *F*_(1,63)_ = 14.238, *p* < 0.001); main effect for genotype: *F*_(1,63)_ = 20.406, *p* < 0.001); main effect for treatment *F*_(1,63)_ = 9.462, *p* < 0.01); interaction for sex × treatment: *F*_(1,63)_ = 12.522, *p* < 0.01); and no other interactions. **(A)** NPC proliferation was significantly increased by EE in male WT mice (WT control males: 4685 ± 353; WT EE males: 7309 ± 547) and male GSK3 KI mice (KI control males: 2906 ± 382; KI EE males: 4915 ± 786). **(B)** NPC proliferation was unchanged by EE in female WT mice (WT control females: 4543 ± 519; WT EE females: 3800 ± 447) and female GSK3 KI mice (KI control females: 2884 ± 338, KI EE females: 3303 ± 499). Values are means ± S.E.M.; *p < 0.05 compared to control WT mice.

We tested if EE affected the impaired NPC proliferation in GSK3 KI mice. Three-way ANOVA condition (genotype × sex × treatment) followed by *post hoc* Bonferroni’s multiple comparison test revealed a main effect for genotype (*F*_(1,63)_ = 20.406, *p* < 0.001), a main effect for sex (*F*_(1,63)_ = 14.238, *p* < 0.001), and a main effect for treatment (*F*_(1,63)_ = 9.462, *p* < 0.01). There was an interaction for sex × treatment (*F*_(1,63)_ = 12.522, *p* < 0.01). EE significantly increased hippocampal NPC proliferation by ~50% in male WT mice (WT control males: 4685 ± 353, WT EE males: 7309 ± 547; *t*_(18)_ = 4.17, *p* < 0.01) (Figure 4A), as previously reported (Komitova et al., 2005; Leal-Galicia et al., 2007; Zhao et al., 2008; Hu et al., 2010; Chakrabarti et al., 2011; Mustroph et al., 2012). Hippocampal NPC proliferation also was increased by EE in male GSK3 KI mice by ~70% (GSK3 KI control males: 2906 ± 382; GSK3 KI EE males: 4915 ± 786, *t*_(14)_ = 2.61, *p* < 0.05). This increase restored proliferation to the level of control male WT mice, but NPC proliferation after EE remained 30% below that of EE-treated male WT mice. Thus, although constitutively active GSK3 impairs basal adult hippocampal NPC proliferation, it does not block enhancement induced by EE in male mice, indicating that the increase induced by EE is independent of GSK3 inhibition by serine phosphorylation.

Female mice differed from male mice in that EE did not significantly increase hippocampal NPC proliferation in female WT mice (WT control females: 4543 ± 519, WT EE females: 3800 ± 447; *t*_(18)_ = 1.09, *p* = 0.29) (Figure 4B), as reported previously (Kempermann et al., 1997; van Praag et al., 1999; Li et al., 2008). EE also did not significantly increase hippocampal NPC proliferation in female GSK3 KI mice (GSK3 KI control females: 2884 ± 338, GSK3 KI EE females: 3303 ± 449; *t*_(13)_ = 0.62, *p* = 0.55), although the small differences after EE eliminated the statistically significant deficit in NPC proliferation compared with female WT mice.

### Effects of EE and TDZD-8 on cognitive tasks in male mice

**Figure 5:**
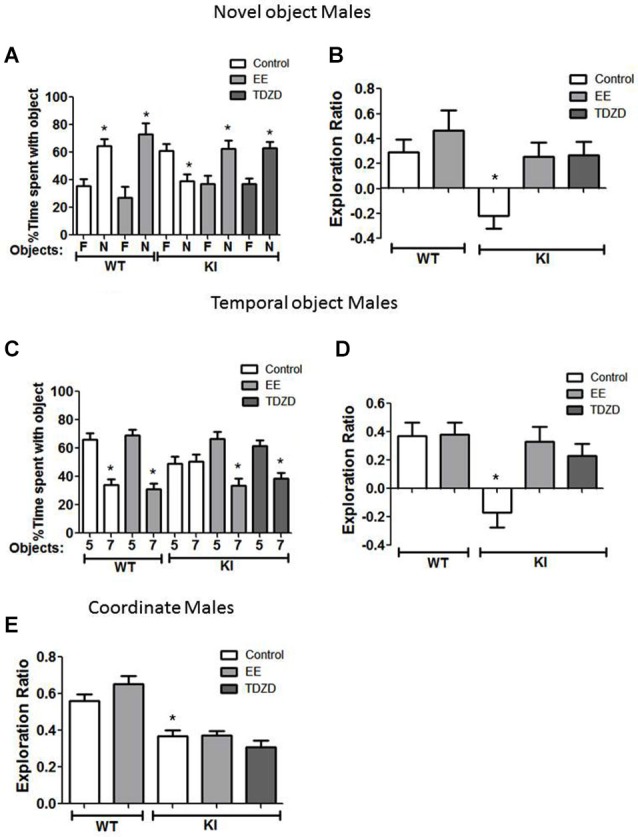
**Environmental enrichment (EE) or TDZD-8 administration improved impaired cognition in male GSK3 KI mice. (A,B)** Effects of EE and TDZD-8 on the performance of male wild-type (WT) (control *n* = 10, EE *n* = 10) and GSK3 KI (control *n* = 12, EE *n* = 14, TDZD-8 *n* = 10) mice on novel object recognition. **(A)** Percent time spent exploring the novel (N) and familiar (F) object. (Student’s *t*-test, *p < 0.05 compared to % time spent with familiar object). **(B)** Exploration ratio (Student’s *t*-test, *p < 0.05 compared to control WT mice). **(C,D)** Effects of EE and TDZD-8 on the performance of male WT (control *n* = 10, EE *n* = 13) and GSK3 KI (control *n* = 12, EE *n* = 13, TDZD-8 *n* = 17) mice on the temporal order task. **(C)** Percent time spent exploring the first (5) and last (7) object presented. (Student’s *t*-test, *p < 0.05 compared to % time spent with object 5). **(D)** Exploration ratio (Student’s *t*-test, *p < 0.05 compared to vehicle-treated WT mice). **(E)** Effects of EE and TDZD-8 on the performance of male WT (control *n* = 10, EE *n* = 13) and GSK3 KI (control *n* = 12, EE *n* = 14, TDZD-8 *n* = 10) mice in the coordinate spatial processing task (Student’s *t*-test, *p < 0.05 compared to controls).

We tested if EE or acute inhibition of GSK3 altered performance of male mice on cognitive tasks. In the novel object recognition test (Figure 5A), EE did not significantly alter the preference of male WT mice for the novel object (control: familiar 36 ± 5 vs. novel 64 ± 5% of total time exploring objects; *t*_(18)_ = 3.99, *p* < 0.01; EE: familiar 27 ± 8 vs. novel 73 ± 8% of total time exploring objects; *t*_(18)_ = 4.00, *p* < 0.01) or the exploration ratio (control 0.29 ± 0.10 vs. EE 0.46 ± 0.16; *t*_(18)_ = 0.90, *p* = 0.38) (Figure 5B). There was a significant effect (genotype × treatment; *F*_(4,55)_ = 4.71; *p* < 0.01) on the exploration ratio. EE normalized the preference of male GSK3 KI mice for the novel object (control: familiar 61 ± 5 vs. novel 39 ± 5% of total time exploring objects; EE: familiar 37 ± 6 vs. novel 63 ± 6% of total time exploring objects; *t*_(26)_ = 3.17, *p* < 0.01), and normalized the exploration ratio (GSK3 KI: control: −0.22 ± 0.10 vs. EE: 0.25 ± 0.11; *t*_(24)_ = 3.09, *p* < 0.01) (Figure 5B). Administration of the GSK3 inhibitor TDZD-8 (5 mg/kg) 1 h prior to testing rescued the impairment in novel object recognition in male GSK3 KI mice (novel 63 ± 4 vs. familiar 37 ± 4% of total time exploring objects; *t*_(28)_ = 4.24, *p* < 0.05) and the impaired exploration ratio increased to a level not significantly different from male WT mice (GSK3 KI TDZD: 0.27 ± 0.11 vs. WT control: 0.30 ± 0.10; *t*_(19)_ = 0.25, *p* = 0.80). We previously reported that novel object recognition in WT mice is unaltered by acute administration of two GSK3 inhibitors, TDZD-8 and VP0.7 (Franklin et al., 2014).

In the temporal order memory task, EE did not alter the performance of male WT mice (Figures 5C,D). There was a significant effect (genotype × treatment; *F*_(4,64)_ = 5.22; *p* < 0.01) on the exploration ratio. In male GSK3 KI mice, EE normalized object preferences to WT levels (Object 5: 67 ± 5, Object 7: 33 ± 5% of total time exploring objects; *t*_(24)_ = 4.48, *p* < 0.01, Figure 5C), which raised the exploration ratio to that of WT mice (control: −0.17 ± 0.11 vs. EE 0.33 ± 0.10; *t*_(23)_ = 3.32, *p* < 0.01). TDZD-8 administration redirected the exploratory behavior of the male GSK3 KI mice towards the object presented first (Object 5: 61 ± 4 vs. Object 7: 39 ± 4% of total time exploring objects; *t*_(32)_ = 3.84, *p* < 0.01) and restored the exploration ratio (control: −0.17 ± 0.11 vs. TDZD 0.23 ± 0.08; *t*_(27)_ = 2.94, *p* < 0.01) to a level that did not significantly differ from male WT mice (*p* = 0.48).

In the coordinate spatial processing task (Figure 5E), there was a significant effect (genotype × treatment; *F*_(4,58)_ = 18.24; *p* < 0.01). EE had no significant effect on the exploration ratio in male WT mice (control 0.56 ± 0.03 vs. EE 0.65 ± 0.04; *t*_(21)_ = 1.53 *p* = 14) or GSK3 KI mice (control 0.37 ± 0.03 vs. EE 0.37 ± 0.02; *t*_(24)_ = 0.09, *p* = 0.93). Interestingly, TDZD-8 treatment also did not restore impaired coordinate spatial processing in male GSK3 KI mice (control 0.37 ± 0.03 vs. TDZD 0.31 ± 0.03; *t*_(22)_ = 1.53, *p* = 0.14).

### Effects of EE and TDZD-8 on cognitive tasks in female mice

**Figure 6:**
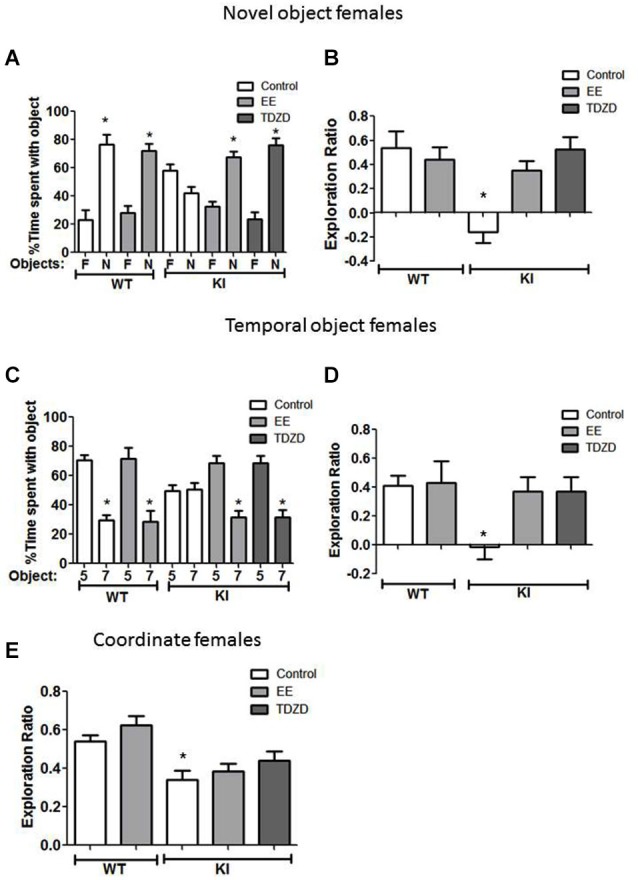
**Environmental enrichment (EE) or TDZD-8 administration improved impaired cognition in female GSK3 KI mice. (A,B)** Effects of EE and TDZD-8 on the performance of female wild-type (WT) (control *n* = 10, EE *n* = 8) and GSK3 KI (control *n* = 9, EE *n* = 9, TDZD-8 *n* = 9) mice on novel object recognition. **(A)** Percent time spent exploring the novel (N) and familiar (F) object. (Student’s *t*-test, *p < 0.05 compared to % time spent with familiar object). **(B)** Exploration ratio (Student’s *t*-test, *p < 0.05 compared to control WT mice). **(C,D)** Effects of EE and TDZD-8 on the performance of female WT (control *n* = 14, EE *n* = 10) and GSK3 KI (control *n* = 10, EE *n* = 14, TDZD-8 *n* = 9) mice on the temporal order task. **(C)** Percent time spent exploring the first (5) and last (7) object presented. (Student’s *t*-test, *p < 0.05 compared to % time spent with object 5). **(D)** Exploration ratio (Student’s *t*-test, *p < 0.05 compared to vehicle-treated WT mice). **(E)** Effects of EE and TDZD-8 on the performance of female WT (control *n* = 24, EE *n* = 16) and GSK3 KI (control *n* = 10, EE *n* = 15, TDZD-8 *n* = 9) mice in the coordinate spatial processing task (Student’s *t*-test, *p < 0.05 compared to controls).

Cognitive behaviors were also examined in female mice after treatment with EE and TDZD-8. There was a significant effect (genotype × treatment; *F*_(4,44)_ = 7.41; *p* < 0.01) between conditions. The impaired novel object recognition in female GSK3 KI mice was repaired by EE (Figure 6A) (familiar 32 ± 4 vs. novel 68 ± 4% of total time exploring objects; *t*_(16)_ = 6.55, *p* < 0.01) and EE increased the exploration ratio (Figure 6B) to a level similar to female WT mice (GSK3 KI EE: 0.35 ± 0.08 vs. WT control: 0.54 ± 0.14; *t*_(17)_ = 1.12, *p* = 0.28). Administration of TDZD-8 restored impaired novel object recognition in female GSK3 KI mice (Figure 6A), resulting in significantly more time spent exploring the novel than familiar object (familiar 24 ± 5 vs. novel 76 ± 5% of total time exploring objects; *t*_(16)_ = 7.41, *p* < 0.01), and significantly increased the impaired exploration ratio (Figure 6B) to a level equivalent to female WT mice (GSK3 KI TDZD: 0.52 ± 0.10 vs. WT control: 0.54 ± 0.14; *t*_(17)_ = 0.07, *p* = 0.95).

With GSK3 KI female mice in the temporal order memory task (Figure 6C), there was a significant effect between experimental conditions (*F*_(4,56)_ = 2.93, *p* < 0.05). EE increased the time female GSK3 KI mice spent exploring the object presented first (Object 5: 69 ± 5 vs. Object 7: 31 ± 5% of total time exploring objects; *t*_(26)_ = 5.44, *p* < 0.01), so the exploration ratio did not differ from that of female WT mice (WT control 0.41 ± 0.07 vs. GSK3 KI EE 0.37 ± 0.10; *t*_(26)_ = 0.30, *p* = 0.77) (Figure 6D). EE did not alter the exploration ratio of female WT mice (control 0.41 ± 0.07 vs. EE 0.43 ± 0.15; *t*_(22)_ = 0.13, *p* = 0.90). After TDZD-8 administration, female GSK3 KI mice explored the first object presented significantly longer than the last object presented (Object 5: 68 ± 5 vs. Object 7: 32 ± 5% of total time exploring objects; *t*_(16)_ = 5.10, *p* < 0.01) and the exploration ratio increased (control −0.01 ± 0.09 vs. TDZD 0.37 ± 0.10; *t*_(17)_ = 2.86, *p* < 0.05) to a value that did not differ from female WT mice (*p* = 0.74).

As with male GSK3 KI mice, there was a significant effect (genotype × treatment; *F*_(4,73)_ =7.31; *p* < 0.01) on the exploration ratio. The impairment in coordinate spatial processing in female GSK3 KI mice (Figure 6E) was unaltered by EE (control 0.34 ± 0.05 vs. EE 0.39 ± 0.04; *t*_(23)_ = 0.71, *p* = 0.49). TDZD-8 treatment caused a slight increase in the exploration ratio of female GSK3 KI mice (GSK3 KI: control 0.34 ± 0.05 vs. TDZD 0.44 ± 0.05; *t*_(17)_ = 0.16, *p* = 0.16), resulting in an exploration ratio that was not significantly different from female WT mice (WT control 0.54 ± 0.03 vs. GSK3 KI TDZD 0.44 ± 0.05; *t*_(31)_ = 1.58, *p* = 0.12).

## Discussion

Hyperactive GSK3 contributes to pathological processes in animal models of a disparate group of diseases that involve impaired cognition, such as Alzheimer’s disease (Martinez and Perez, 2008; Avila et al., 2010), mood disorders (O’Brien and Klein, 2009; Jope, 2011), and Fragile X syndrome (Mines and Jope, 2011). Conversely, administration of GSK3 inhibitors ameliorates cognitive impairments in multiple conditions (King et al., 2014). However, it is not clear if cognitive impairments result directly from hyperactive GSK3 or from actions of GSK3 promoting pathological processes, such as promoting protein aggregates in mouse models of Alzheimer’s disease (King et al., 2014). Here we used GSK3 KI mice, which express constitutively active GSK3, to test if increased GSK3 activity is sufficient to impair cognitive processes in mice without associated pathology (McManus et al., 2005; Eom and Jope, 2009). We found that GSK3 KI mice have severe deficits in three cognitive assessments, novel object recognition, temporal order memory, and coordinate spatial processing, that were as severe as recently found in the mouse model of Fragile X syndrome (King and Jope, 2013; Franklin et al., 2014). These cognitive impairments in GSK3 KI mice affected both sexes, and were significantly improved by housing mice in EE and by acute treatment with a GSK3 inhibitor. Both sexes of GSK3 KI mice also displayed impaired adult hippocampal NPC proliferation, but changes effected by EE and TDZD-8 indicate that deficits in NPC proliferation did not cause the impairments in novel object recognition or temporal ordering.

The finding that female GSK3 KI mice exhibit impaired hippocampal NPC proliferation compared to female WT mice extends previous findings indicating that GSK3 can regulate this process. These include reports that NPC proliferation is impaired by constitutively active GSK3 in male GSK3 KI mice (Eom and Jope, 2009), molecular deletion of GSK3 in mouse neural progenitors increased neurogenesis (Kim et al., 2009), and neurogenesis is increased by treatment with lithium or other drugs that inhibit GSK3 (Chen et al., 2000; Hashimoto et al., 2003; Silva et al., 2008; Wexler et al., 2008; Kim et al., 2009; Mao et al., 2009; Serenó et al., 2009; Fiorentini et al., 2010; Guo et al., 2012; Morales-Garcia et al., 2012; Contestabile et al., 2013). Housing male, but not female, WT mice in EE led to increased hippocampal NPC proliferation. These results are in accordance with previous reports that EE increases hippocampal neurogenesis in male WT mice (Komitova et al., 2005; Leal-Galicia et al., 2007; Duman et al., 2008; Greenwood and Fleshner, 2008; Zhao et al., 2008; Hu et al., 2010; Chakrabarti et al., 2011; Jha et al., 2011), but not in female mice (Kempermann et al., 1997; van Praag et al., 1999; Brown et al., 2003; Westenbroek et al., 2004; Kobilo et al., 2011). Extending the analysis to GSK3 KI mice demonstrated that EE increased hippocampal NPC proliferation in male GSK3 KI mice by the same percentage as in male WT mice. This demonstrates that inhibitory serine-phosphorylation of GSK3 is not required for EE to enhance hippocampal NPC proliferation. This differs from the requirement for serine-phosphorylation of GSK3 for treatment with lithium and fluoxetine to increase hippocampal NPC proliferation (Eom and Jope, 2009). Hippocampal NPC proliferation was not affected by EE in female GSK3 knockin mice, demonstrating that there are sex differences in the influence of the environment on hippocampal NPC proliferation in both WT mice and in mice in which GSK3 is hyperactive. The differential effects of EE on hippocampal NPC proliferation in male and female GSK3 KI mice provided a mechanism to determine if this process is required for EE-induced improvements in performance in several cognitive tasks without the need to use toxic interventions to ablate NPC proliferation.

Novel object recognition is a measure of recognition memory that assesses the ability of mice to recognize previously encountered objects by determining their propensity to explore a novel object more than a familiar object (Antunes and Biala, 2012). Novel object recognition has been reported to be a task both independent of the hippocampus (Winters et al., 2004, 2008; Winters and Bussey, 2005; Dere et al., 2007; Hunsaker and Kesner, 2008) and hippocampal-dependent (Clark et al., 2000; Hunsaker et al., 2008; Dewachter et al., 2009; Fortress et al., 2013; Franklin et al., 2014). Novel object recognition was severely impaired in male GSK3 KI mice compared to male WT mice. This finding extends a previous report that male GSK3β KI mice display impaired novel object recognition (Dewachter et al., 2009). Upon assessment of novel object recognition in female WT mice, we found them unable to differentiate between the novel and familiar objects in the paradigm used with male mice. Previous comparisons of sexes in novel object recognition reported no differences (Benice et al., 2006), or superior performance of females (Podhorna and Brown, 2002; Sutcliffe et al., 2007) or of males (Frick and Gresack, 2003). Because of this complication in assessing female mice, we used a simpler procedure for female mice than for male mice in which female WT mice successfully displayed novel object recognition. With this protocol, we found that female GSK3 knockin mice displayed a severe deficit in novel object recognition compared with female WT mice. Previous studies have reported that performance on the novel object recognition test was enhanced in parallel with increased hippocampal NPC proliferation in response to exercise (Bechara and Kelly, 2013), by lithium treatment of mice modeling Down syndrome (Contestabile et al., 2013), or by EE in rat models of global ischemia (Kato et al., 2014) or type 1 diabetes (Piazza et al., 2011). Conversely, concurrent impairments in novel object recognition and NPC proliferation have been reported after a number of insults (Graciarena et al., 2010; Nam et al., 2013; Chen et al., 2014; Greene-Schloesser et al., 2014). We found that housing mice in EE improved novel object recognition in both male and female GSK3 KI mice. However, EE only increased hippocampal NPC proliferation in male GSK3 KI mice, not female GSK3 KI mice, indicating that EE-induced improved novel object recognition can occur independently of increased NPC proliferation. Furthermore, treatment with the GSK3 inhibitor TDZD-8 for 1 h, a time too short to functionally alter NPC proliferation, also improved novel object recognition in both male and female GSK3 KI mice. Thus, constitutively active GSK3 impaired both novel object recognition and hippocampal NPC proliferation in both male and female GSK3 KI mice, but novel object recognition could be repaired independently of NPC proliferation. We suggest that impaired novel object recognition in GSK3 KI mice may be linked to deficient LTP, which requires inhibition of GSK3 (Hooper et al., 2007; Peineau et al., 2007; Dewachter et al., 2009; Franklin et al., 2014).

The temporal order task assesses episodic-like memory in mice based on their preference to explore an object that was presented earlier in time than an object that was more recently explored (Dere et al., 2005a,b; Hoge and Kesner, 2007), and is reported to be dependent on the hippocampal CA1 region (Kesner et al., 2004, 2005; Hunsaker et al., 2006; Hoge and Kesner, 2007). Temporal order recognition was significantly impaired in both male and female GSK3 KI mice compared with gender-matched WT mice. EE ameliorated impaired temporal order recognition in both male and female GSK3 KI mice. Improvements in temporal order object recognition were unrelated to increased hippocampal NPC proliferation since the latter occurred only in male, but not female, GSK3 KI mice, and acute TDZD-8 pretreatment restored temporal order recognition in both sexes. Normalization by acute treatment with GSK3 inhibitors of impaired temporal order recognition in a mouse model of Fragile X syndrome in which GSK3 is hyperactive was previously suggested to involve improved function of the hippocampal trisynaptic circuit, although this remains to be tested (King and Jope, 2013; Franklin et al., 2014).

The coordinate spatial processing task assesses hippocampus-dependent spatial memory in mice based on their ability to recognize a change in the metric of the environment by comparing the exploration time between the end of the habituation period and a period when the distance between objects was changed (Galani et al., 1998; Goodrich-Hunsaker et al., 2005, 2008). Coordinate spatial memory was severely impaired in male and female GSK3 KI mice compared with gender-matched WT mice. EE did not modify coordinate spatial memory in any group of mice, suggesting that this process is independent of hippocampal NPC proliferation, which was increased by EE in male GSK3 KI mice. The impairment that male and female GSK3 KI mice displayed in this spatial task also was not improved by acute TDZD-8 pretreatment. Previous studies have described concurrent enhanced spatial memory and increased NPC proliferation after EE (Okun et al., 2010; Li et al., 2011; Kato et al., 2014), and concurrent impaired spatial memory and impaired NPC proliferation (Umka et al., 2010; Li et al., 2013; Nam et al., 2013; Ambrée et al., 2014; Chen et al., 2014; Greene-Schloesser et al., 2014; Valero et al., 2014). However, studies of the dependence of cognitive behaviors on NPC proliferation have often relied on correlations or have used NPC-ablating approaches, whereas in the present study EE was used to increase NPC proliferation. The results found here agree with previous findings that adult hippocampal neurogenesis is not obligatory for certain forms of learning and memory (Jaholkowski et al., 2009; Groves et al., 2013), and with their suggestion that studies using NPC-ablating approaches, such as irradiation, drug treatments, or mutant mice, may be confounded by other actions that could interfere with the interpretation of the results.

Interactions between genetics and the environment may have tremendous influences on susceptibilities to cognitive impairments in many diseases. However, much still remains to be learned about factors that mediate differences in susceptibility and severity of cognitive impairments. This study examined the combinatorial effects of genetics (gender, hyperactive GSK3) and the environment (EE) to determine factors that mediate differences in cognition in mice. Expression of maximally active GSK3 in GSK3 KI mice was sufficient to impair performance in three cognitive tasks, and these impairments were equivalent in male and female GSK3 KI mice. Thus, hyperactive GSK3, which occurs in several conditions displaying impairments in cognition, is sufficient to impair cognition, further supporting previous reports that GSK3 inhibitors may alleviate cognitive impairments in a number of conditions (King et al., 2014).

## Conflict of interest statement

The authors declare that the research was conducted in the absence of any commercial or financial relationships that could be construed as a potential conflict of interest.
